# Abnormal activity of transcription factors gli in high-grade gliomas

**DOI:** 10.1371/journal.pone.0211980

**Published:** 2019-02-07

**Authors:** Andrey Volnitskiy, Tatiana Shtam, Vladimir Burdakov, Roman Kovalev, Alexander Konev, Michael Filatov

**Affiliations:** 1 Petersburg Nuclear Physics Institute named by B.P. Konstantinov of National Research Centre "Kurchatov Institute", Gatchina, Russia; 2 N.N. Petrov National Medical Research Center of Oncology, St. Petersburg, Pesochnyj, Leningradskaya, Russia; Macau University of Science and Technology, MACAO

## Abstract

Malignant transformation is associated with loss of cell differentiation, anaplasia. Transcription factors gli, required for embryonic development, may be involved in this process. We studied the activity of transcription factors gli in high-grade gliomas and their role in maintenance of stem cell state and glioma cell survival. 20 glioma cell lines and a sample of a normal adult brain tissue were used in the present study. We found the expression of gli target genes, including *GLI1* and *FOXM1*, in all tested glioma cell lines, but not in the normal tissue. Interestingly, the expression of gli target genes in some glioma cell lines was observed together with a high level of their transcriptional repressor, Gli3R. Knockdown of *GLI3* in one of these lines resulted in decrease of gli target gene expression. These data suggest that Gli3R does not prevent the gli target genes transcription, and gli3 acts in glioma cells more as an activator, than a repressor of transcription. We observed that gli regulated the expression of such genes, as *SOX2* or *OCT4* that maintain stem cell state, and *TET1*, involving in DNA demethylation. Treatment with GANT61 or siRNA against *GLI1*, *GLI2*, or *GLI3* could result in complete glioma cell death, while cyclopamine had a weaker and line-specific effect on glioma cell survival. Thus, the gli transcription factors are abnormally active in high-grade gliomas, regulate expression of genes, maintaining the stem cell state, and contribute to glioma cell survival.

## Introduction

High-grade gliomas are invasive, rapidly progressive brain tumors that poorly respond to standard therapies. Malignant transformation, leading to glioma appearance, is associated with loss of cell differentiation, anaplasia. Activation of mechanisms, maintaining stem cell state, is a possible cause of this process. The Sonic Hedgehog (Shh) signaling pathway and its downstream transcription factors gli are considered as one of these mechanisms [1–3].

The gli1, gli2 and gli3 proteins are required for vertebrate embryonic development, including the formation of nervous system. These transcription factors contain zinc finger motifs in their DNA-binding regions and recognize the GACCACCCA consensus sequence on promoters of their target genes [4, 5]. The gli transcription factors regulate an expression of a wide range of genes, involving in cell cycle and cell differentiation, including *CDC2*, *hTERT*, *IRS1*, *FOXM1* and *BMI1* [6–10]. The *GLI1* and *PTCH* genes, encoding the components of the Shh signaling pathway, are also canonical gli target genes.

In the cytoplasm, gli proteins form a complex with Sufu, retaining them in inactive state [11, 12]. This complex dissociates at the tip of primary cilia [12–14]. However, protein kinase A (PKA), located at the base of the primary cilium, phosphorylates gli, preventing the ciliary localization of gli2 and gli3 [15, 16] and inactivating gli1 [3, 17, 18]. In addition, PKA and GSK3β determine a partial cleavage of gli2 and gli3 to turn them into transcription repressors, which directionally suppress transcription of gli target genes [19–22]. The ligand Shh associates with the receptor Ptch, leading to the accumulation of molecules that activate the Smo protein [23, 24]. Smo accumulates in the primary cilium [25] and inhibits the activity of adenylate cyclase and, consequently, PKA [26–28]. In the result, gli proteins accumulate at the tip of the cilium [13, 14], where they dissociate from Sufu, and translocate to the nucleus as transcription activators [12, 14].

Previously, we detected that glioma cells possess the abnormal expression of genes, involved in maintenance of stem cell state, including *SOX2* [29]. We noticed that *SOX2* expression can be regulated by gli [30, 31]. These findings suggest a possible involvement of gli in the development of high-grade gliomas. In this work, we studied the activity of the gli transcription factors in high-grade gliomas and their role in maintenance of stem cell state and survival of glioma cells.

## Materials and methods

### Glioma cell lines and a normal adult brain tissue

Glioma cell lines A-172 and T98G from the cell culture collection of the Institute of Cytology RAS, 18 primary cultures, derived from surgical samples of one anaplastic astrocytoma (GCL 6) and 17 multiform glioblastomas (WHO grade III and IV) [29], and also a morphologically normal adult brain tissue, obtained from one of glioma samples, were used in the present study.

All procedures for obtaining biopsies as a result of elective surgery for medical reasons were performed by physicians at the Polenov Neurosurgical Institute. All patients provided informed consent. The protocol and design of the study were approved by the Academic Council and Ethics Committee of the Polenov Neurosurgical Institute.

Cells were cultured in DMEM/F-12 (1:1) medium, containing L-glutamine and supplemented with 2.5 or 10% fetal bovine serum (BioloT). The medium, containing 2.5% serum, was used for incubation with inhibitors or siRNA, and serum was added only 90 minutes after the addition of inhibitors or siRNA.

### Total RNA extraction and Real Time Quantitative RT-PCR (TaqMan)

Total RNA was extracted from about two million cells using Aurum total RNA minikit (BioRad) with the addition of DNase I for degradation of genomic DNA. Reverse transcription was performed with iScript cDNA Synthesis Kit (Bio-Rad) according to the manufacturer's protocol.

Real Time Quantitative RT-PCR was performed on the thermocycler DT-322 (DNA-Technology) in 50 μl of the reaction mixture for 45 cycles. The reaction mixture contained 1 mM of magnesium chloride, 250 μM of each dNTP, 2.5 units of Taq polymerase (Silex), 15 pmol of forward and reverse primers, 15 pmol of a fluorescently labeled probe (Syntol) and 2 μg of cDNA. Each cycle included DNA denaturation at 95˚C for 15 seconds and primer annealing and DNA amplification at 60˚C for 1 minute. The mRNA levels of tested genes were normalized on *GAPDH* mRNA levels. Total RNA samples without reverse transcription were to control cDNA contamination by genomic DNA. Sequences of primers and fluorescently labeled probes are shown in the Table 1.

**Table 1:** Sequences of primers and fluorescently labeled probes.

	Forward primer	Reverse primer	Probe (FAM-BHQ1)
*GLI1*	aactccacaggcatacagga	tggatgtgctcgctgttgat	agaagcgtgagcctgaatctgtg
*GLI2*	tggctgacctcaaggaagat	ggatgtgctcgttgttgatg	tctatgagaccaactgccactgg
*GLI3*	agtgaagtgctccactcgaa	cactcgatgttgaaggttcc	tccagcaccacttctaatgaggat
*FOXM1*	tggcagaactctgtgtctga	ttgtggcggatggagttctt	aattcgccatcaacagcactgaga
*BMI1*	ttacctggagaccagcaagt	attggcagcatcagcagaag	gtccaagttcacaagaccagacc
*OCT4*	aagctggagaaggagaagct	cagatggtcgtttggctgaa	actcgagcaatttgccaagctc
*SOX2*	atgagagagatcctggactt	tcgcttggagactagctct	tggaccttgtatagatctggagga
*TET1*	cagtcagaagccttctgaca	ttggatttggctacgaccag	gaacccagagtccttaacctgca
*GAPDH*	catgggtgtgaaccatgagaa	ggtcatgagtccttccacgat	aacagcctcaagatcatcagcaatgcct

### Western blotting of the Gli1, Gli3 and Tet1 proteins

Cells were incubated at 4°C for 30 minutes with a lysis buffer (10 mM Tris-HCl pH 7.4, 0.1% Triton X-100, 5 mM MgCl_2_, 20 mM β-mercaptoethanol, 5 U/ml of DNase I, Protease Inhibitor Cocktail (Pierce) for Gli1 or Gli3; and 7M urea, 2M thiourea, 4% CHAPS, 5 mM PMSF, 1% DTT for Tet1). Total protein levels were determined using Bradford reagent for equal application on electrophoresis tracks. The protein samples were diluted in Laemmli buffer, subjected to SDS-PAGE (10% for Gli1 or Gli3 and 6% for Tet1), containing 0.1% SDS, and transferred to the PVDF membrane (Thermo Scientific). Immunoblotting was performed according to the Blue Dry Western protocol [32]. GAPDH was detected as a loading-control. Mouse monoclonal antibodies to Gli1 (Abnova) or GAPDH (Merck Millipore), at dilutions of 1:300 and 1:500, respectively, as well as rabbit polyclonal antibodies to Gli3 (Abcam) or Tet1 (GeneTex), at dilutions of 1:300 and 1:600, respectively, were used as primary antibodies. Horseradish peroxidase-conjugated goat antibodies against mouse or rabbit immunoglobulins from Sigma were used as secondary antibodies in dilutions of 1:2000 and 1:5000, respectively. Chemiluminescent detection of the protein bands was performed with Clarity Western ECL Blotting Substrate (Bio-Rad). Densitometry analysis was done by TotalLab quant software.

### Treatment with gli inhibitors and knockdown of gli

Cyclopamine inactivates gli through Smo inhibition, while GANT61 directly prevents gli binding to DNA. Glioma cells were treated with 10, 20, 40 and 80 μM of cyclopamine, 20 and 40 μM GANT61 (Tocris Bioscience), or 0.1 pmol of siRNA against *GLI1*, *GLI2* or *GLI3* (ON-TARGETplus SMARTpool, Dharmacon). The siRNA was introduced into the cells using Lipofectamine (Thermo Scientific DharmaFECT) according to the manufacturer's protocol. Lipofectamine alone or previously tested siRNA against *TET2* that does not affect glioma cell survival were used to control the possible non-specific transfection toxicity.

Cells were incubated in a 25 ml flask (5*10^5^ cells per a flask) for 48 hours to observe the expression of gli target genes, and on a 24-well plate (2*10^4^ cells per a well) for 120 hours (when cells in the control were reached the monolayer) to study the cell survival. The plates had been stained with the crystal violet for visualization of the cell survival.

In addition, MTS-assay was used to study cell survival. Cells were incubated in a 96-well plate (2*10^4^ cells per a well) for 48 hours with GANT61 or cyclopamine. HeLa (from the Institute of Cytology RAS) was used as a negative control. MTS-reagent (Promega) was added to the plate according to the manufacturer’s protocol. The absorbance at 490nm was detected using EnSpire Multimode Plate Reader (PerkinElmer, USA).

## Results

### The expression of *GLI1*, *GLI2*, *GLI3* and their target genes in glioma cell lines and the normal adult brain tissue

**Fig 1:**
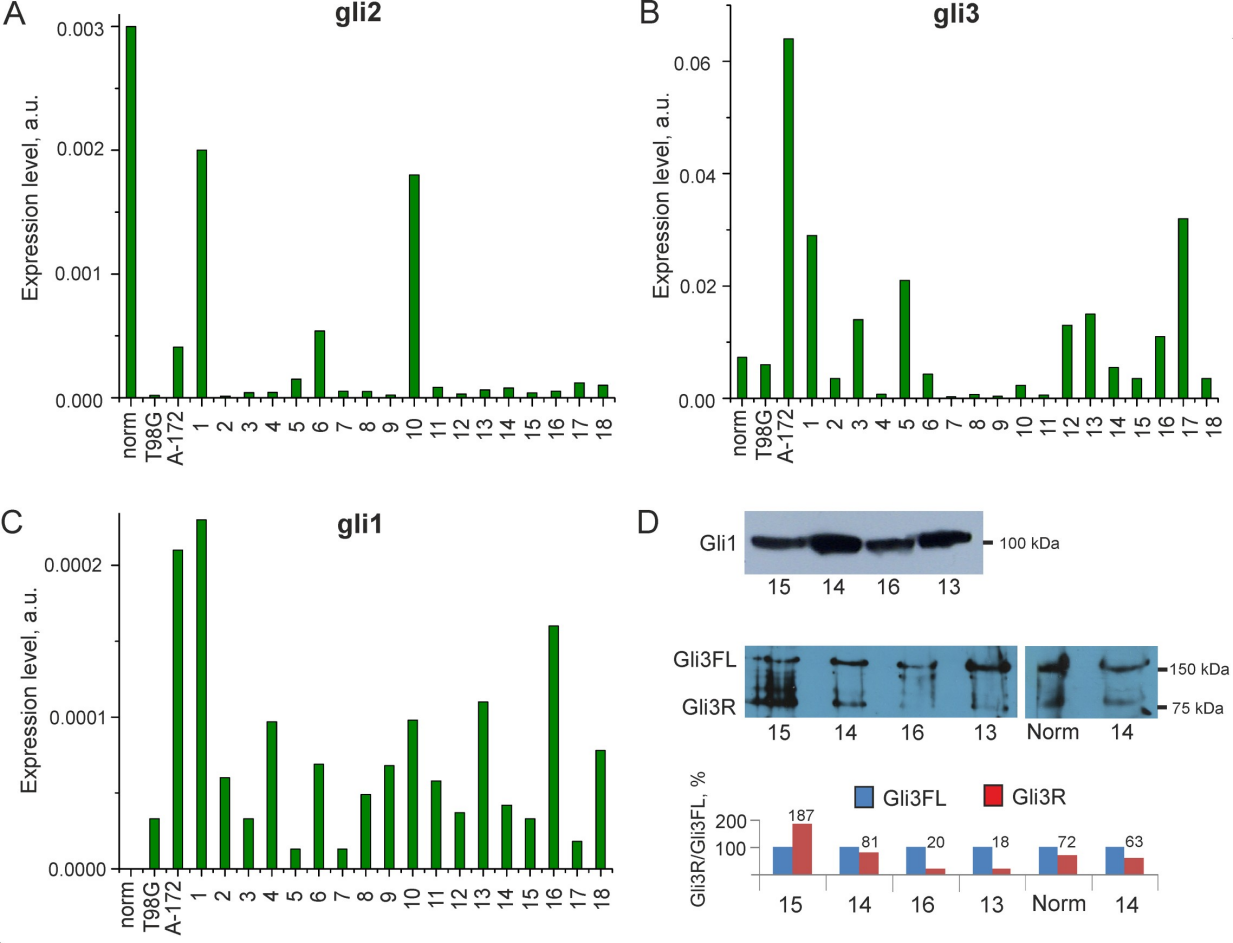
Expression of *GLI* genes in glioma cells and norm. The mRNA levels of (**A**) *GLI2*, (**B**) *GLI3*, and (**C**) their target gene *GLI1*, normalized to that of *GAPDH*, in glioma cell lines and the normal adult brain tissue. (**D**) Western blotting of the Gli1 and Gli3 proteins; detection and densitometry of the Gli3R/Gli3FL ratio in GCLs 13, 14, 15, 16 and the normal adult brain tissue. The histogram numbers indicate the percentage of GLI3R.

**Fig 2:**
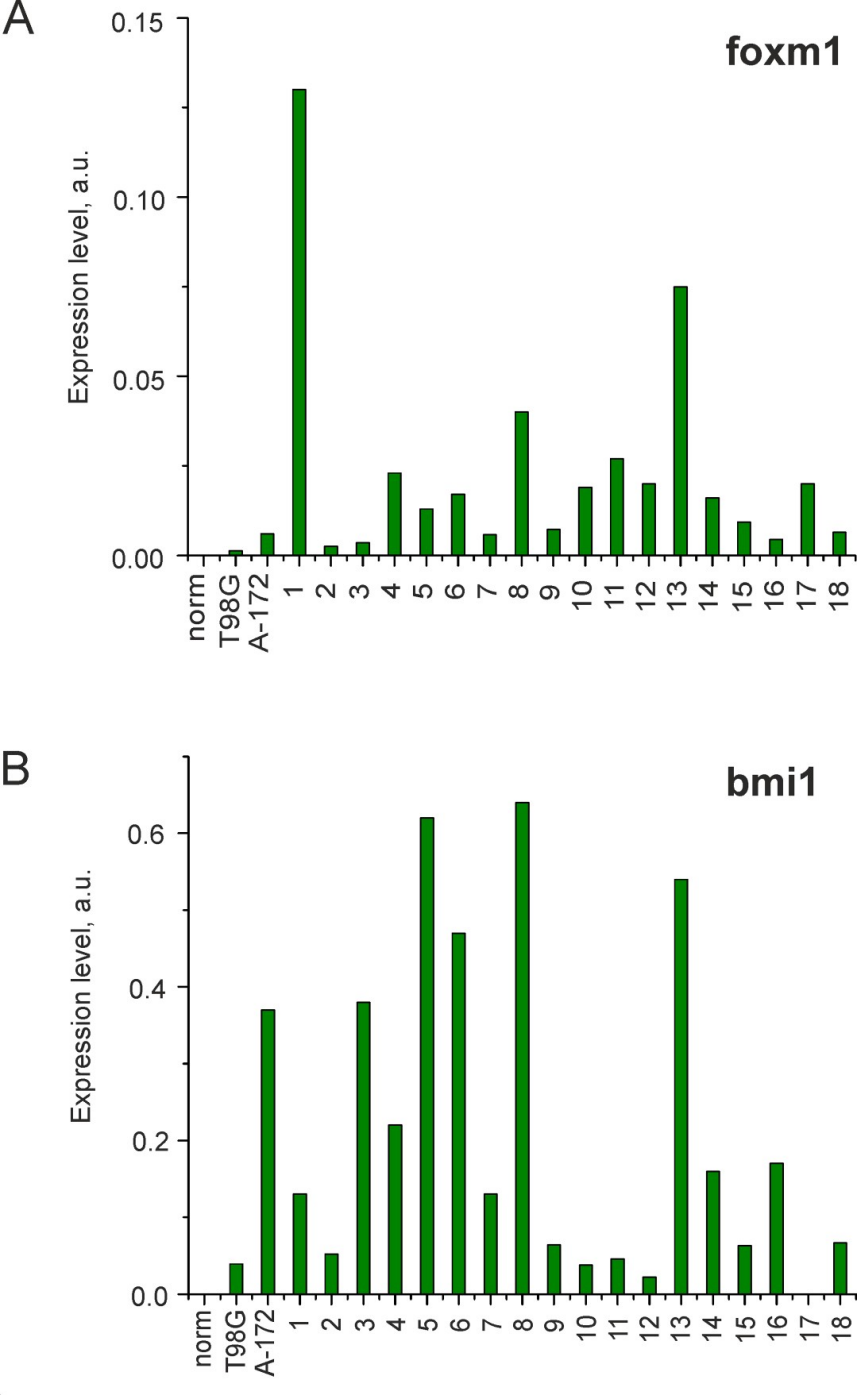
Expression of gli target genes in glioma cells and norm. The mRNA levels of (**A**) *FOXM1* and (**B**) *BMI1*, normalized to that of *GAPDH*, in glioma cell lines and the normal adult brain tissue.

The expression of *GLI1*, *GLI2*, *GLI3* and their target genes *FOXM1* and *BMI1* [9, 10] was analyzed by RT-qPCR for 20 glioma cell lines and a normal adult brain tissue. The expression of *GLI2* and *GLI3* was detected in all GCLs, as well as the normal adult brain tissue (Fig 1A and 1B). However, the expression of gli target genes, including *GLI1*, *FOXM1* and *BMI1*, was found in GCLs only (20, 20 and 19 of 20 cases, respectively), but not in the normal adult brain tissue (Fig 1C, Fig 2A and 2B).

### The expression of gli target genes in glioma cells can be observed together with a high level of their transcriptional repressor, Gli3R

We performed Western-blotting of gli1 and gli3 proteins for four primary glioma cell lines, but the data were unexpected (Fig 1D). The Gli1 protein, indicating gli activity, was found in all tested GCLs (Fig 1D). However, some of these lines (GCLs 14 and 15) had a high level of the gli3 truncated form, Gli3R, which is a transcriptional repressor of gli target genes. The ratio of the Gli3R (83 kDa) and Gli3FL (190 kDa) levels in GCL 14 was similar to that in the normal adult brain tissue, where expression of gli target genes was not detected. In GCL 15 this ratio was even higher than that in the normal. The densitometry data are presented in the Fig 1D.

### The gli transcription factors regulate the expression of genes, maintaining the stem cell state

**Fig 3:**
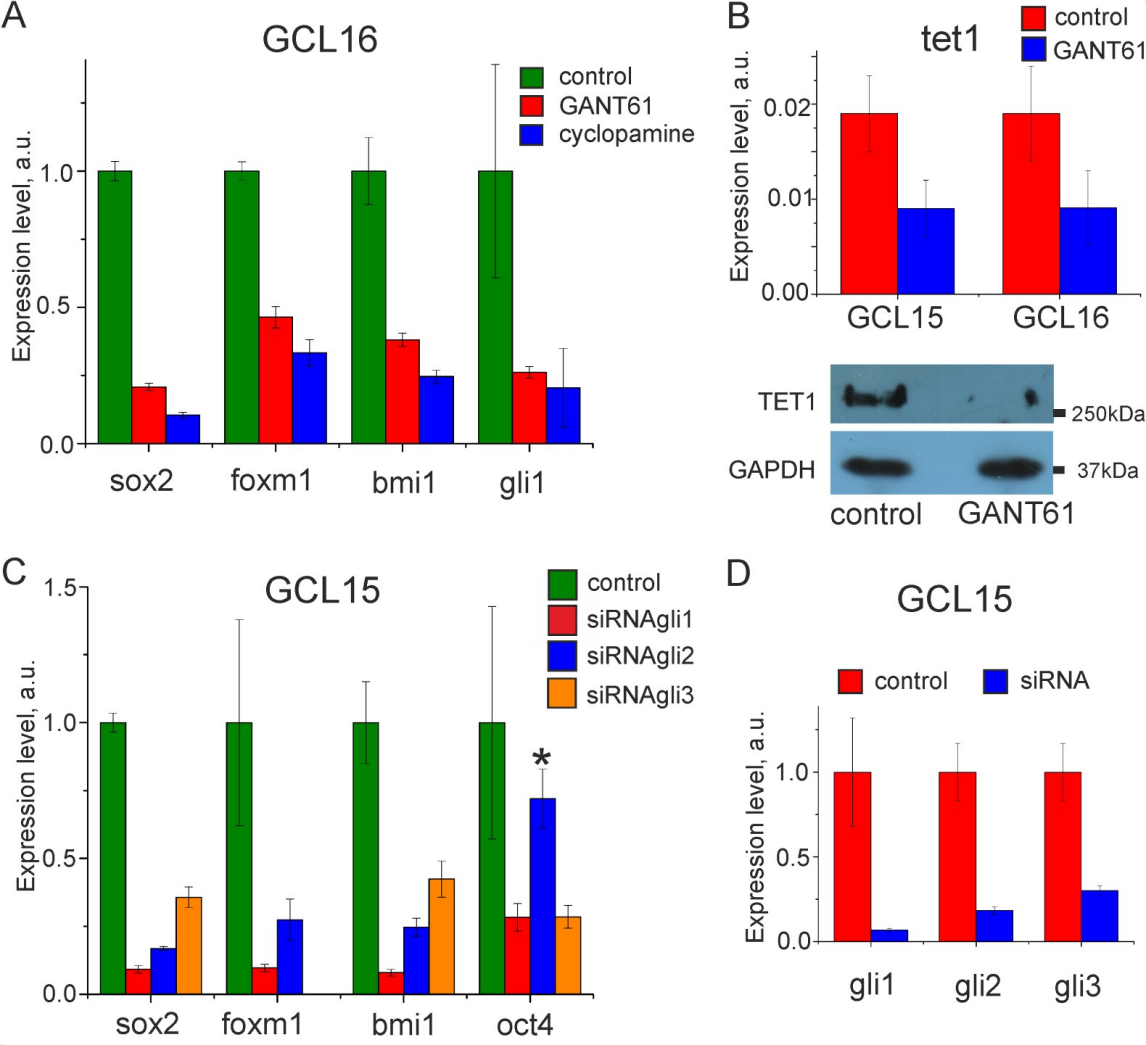
Expression of target genes after gli inactivation in glioma cells. (**A**) Normalized to control mRNA levels of *SOX2*, *FOXM1*, *BMI1* and *GLI1* after treatment of GCL 16 with 20μM GANT61 or 10μM cyclopamine. (**B**) Analysis of *TET1* expression in some glioma cell lines, treated with 40μM GANT61: *TET1* relative mRNA levels (GCLs 15 and 16, up panel) and Western-blotting detection of Tet1 (GCL 15, down panel). (**C**) Normalized to control mRNA levels of *SOX2*, *FOXM1*, *BMI1* and *OCT4* after knockdown of *GLI1*, *GLI2* and *GLI3* in GCL 15. (**D**) Knockdown efficiency: normalized to control mRNA levels *GLI1*, *GLI2* and *GLI3*, after treatment with siRNA against *GLI1*, *GLI2* or *GLI3*, respectively. The samples of untreated or non-specifically transfected cells are named as a control. Error bars on the all histograms indicate the standard deviation of at least three independent experiments. Statistical significance of observed difference between two groups (treated cells vs. control) was determined by ANOVA (in all histograms p < 0.05, except for *p > 0.05 –insignificant).

The treatment of glioma cells with cyclopamine, GANT61 or siRNA against *GLI1*, *GLI2*, or *GLI3* significantly decreased the expression of *GLI1*, *FOXM1*, *BMI1*, *SOX2*, and *OCT4*, involving in the maintenance of the stem cell state. The histograms on the Fig 3 demonstrate the reduction in mRNA levels of these genes upon incubation with the inhibitors (Fig 3A) or knockdown of *GLI1*, *GLI2*, or *GLI3* (Fig 3C). In addition, the treatment of glioma cells with GANT61 resulted in decrease of mRNA and protein levels of tet1, involved in DNA demethylation and cell reprogramming (Fig 3B).

Interestingly, *GLI3* knockdown reduced the expression of gli target genes, despite the high level of their transcriptional repressor Gli3R (Fig 3C). According these data, in glioma cells, gli3 acted more as an activator, than repressor of transcription.

### Glioma cells are sensitive to immediate gli inactivation by GANT61 or knockdown, but are more resistant to the Smo inhibitor, cyclopamine

**Fig 4:**
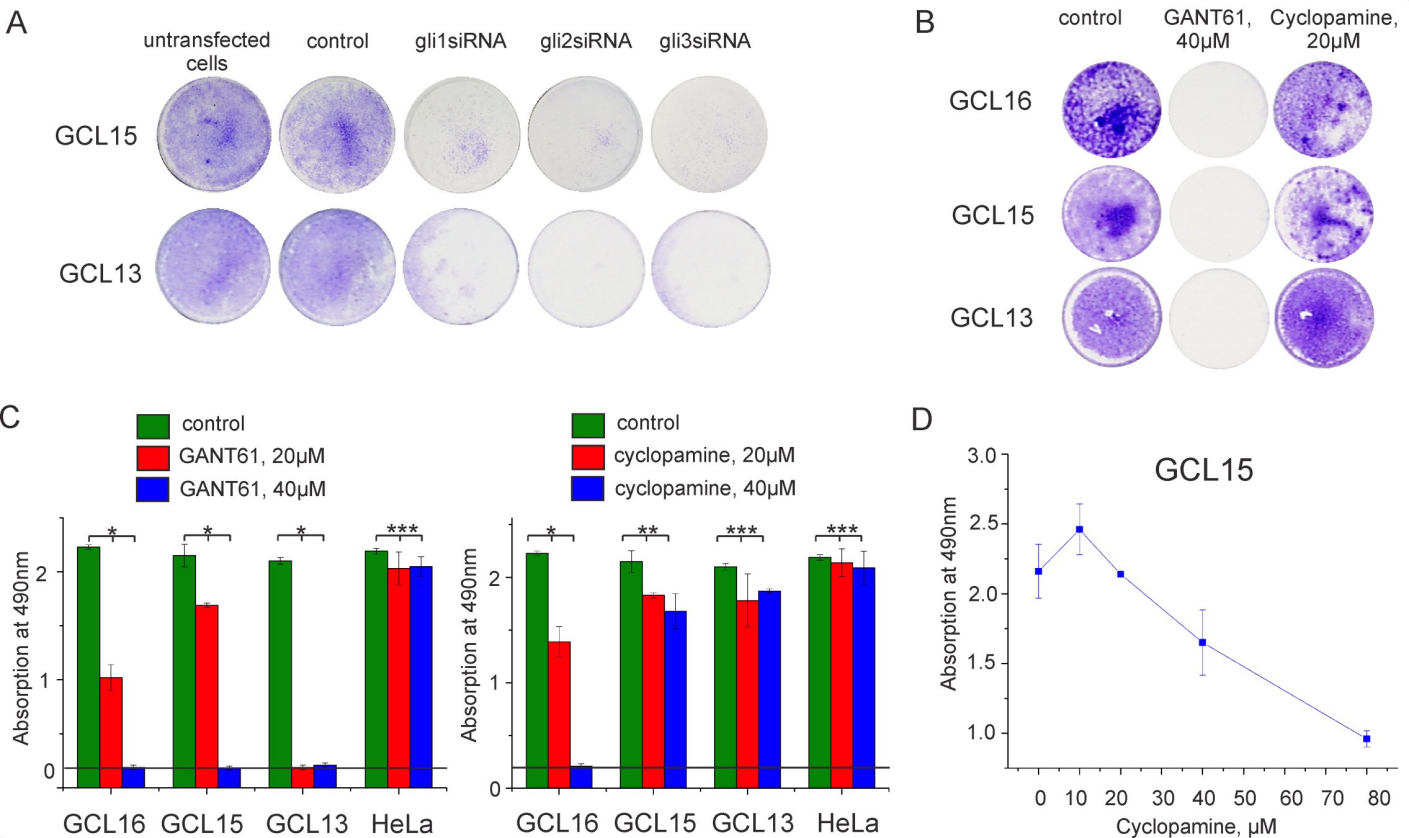
Glioma cell survival after gli inactivation. (**A, B**) Cell survival after treatment of some GCLs with siRNA against *GLI1*, *GLI2*, or *GLI3*, 40μM of GANT61 or 20μM of cyclopamine. The samples of untreated cells are named as a control (**C**) MTS-test after treatment of some GCLs and HeLa with 20 and 40μM of cyclopamine or GANT61. Horizontal lines on the histograms indicate the background levels. Error bars on the histograms indicate the standard deviation of at least three independent experiments. Statistical significance of observed difference between two groups (treated cells vs. control) was determined by ANOVA (*p < 0.005, **p < 0.05, ***p > 0.05 –insignificant) (**D**) MTS-test after treatment of GCL 15 with 10, 20, 40 and 80μM of cyclopamine.

The incubation of glioma cells with siRNA against *GLI1*, *GLI2*, or *GLI3* resulted in death of glioma cells (Fig 4A). Thus, each of the gli transcription factors can contribute to glioma cell survival. Inhibitors GANT61 and cyclopamine acted differently on glioma cells and did not affect the survival of HeLa cells used as a negative control. The action of GANT61 depended on its concentration and could lead to complete glioma cell death already at 20 or 40μM per 2*10^4^ cells (Fig 4B and 4C). Cyclopamine acted on different GCLs with different efficiencies and did not lead to the complete cell death of some lines even in concentrations of 40 or 80μM per 2*10^4^ cells (Fig 4B, 4C and 4D). For comparison, the expression of gli target genes was reduced at 10 μM of cyclopamine (Fig 3A). This resistance of glioma cells to cyclopamine indicates that the total activity of gli can be maintained both through the Smo protein and independently of it.

## Discussion

The glioma-associated oncogenes, gli, were first isolated from human glioblastoma cells in 1987 [4, 5]. However, first studies resulted in conclusion that gli expression is a rare in neural tumors and associated mainly with DNA amplification [33, 34]. The role of gli in malignant gliomas has been a subject of scientific researches only since 2001 [1, 35].

Thus, the expression of the components of the Shh signaling pathway, including *GLI1*, *GLI2*, *GLI3*, *PTCH*, and *SMO*, was found to be common in various tumors of the nervous system, including gliomas [1, 36], and correlated with a poor prognosis of patient survival [37]. It was shown that gli maintain the chromatin active state in the promotors of their target genes in glioma cells [38], but are not active in a normal adult brain [1, 2, 39]. It was revealed that gli promote glioma cell mobility [40, 41]. The splice variant of gli1, tgli1, specifically regulating the migration of tumor cells and angiogenesis, was discovered in multiform glioblastomas [42–44]. It was shown that gli activity is associated with glioma cells, containing stem cell markers [1, 2, 39, 45, 46]. It was found that gli regulate the expression of *OLIG2*, *SOX2*, *BMI1*, and *NANOG* genes that determine the stem cell state [6, 10, 47, 48]. Inhibitors of Shh/gli were shown to suppress the growth of glioma cells and significantly reduced their resistance to temozolomide [39, 49–51] or radiation therapy [45, 52].

It has been shown, that glioma cells are regulated by the Shh signaling pathway, however, normal astrocytes and endothelial cells of blood vessels, sprouting into the tumor, but not the tumor cells themselves, are the source of the ligand [2, 47, 53, 54]. At the same time, activation or inhibition of the Shh signaling pathway often did not affect gli activity or tumor cell growth in multiform glioblastomas [2, 46, 55–57]. Thus, the question of the regulation of gli activity in high-grade gliomas is still open.

We found the expression of *GLI1*, *GLI2*, *GLI3* and their target genes *FOXM1* and *BMI1* in all tested glioma cell lines, whereas the normal adult brain tissue contained only the expression of *GLI2* and *GLI3*. Thus, our data suggest that transcription factors gli are active in gliomas, but not in a normal adult brain tissue. Treatment of glioma cells with the inhibitors or siRNA against *GLI1*, *GLI2*, or *GLI3* caused the significant decrease in the expression of gli target genes, including *GLI1*, *FOXM1* and *BMI1*. We confirmed that in high-grade gliomas, gli maintain the expression of genes, determining the stem cell state, such as *OCT4* or *SOX2* [58], and discovered that gli regulate the expression of TET1 dioxygenase, involved in DNA demethylation and cell reprogramming [59–61].

According to our data, treatment of glioma cells with GANT61 or siRNA against *GLI1*, *GLI2*, or *GLI3* resulted in their death, indicating, that gli contribute to glioma cells survival. However, cells of the same lines were more resistant to cyclopamine, which inhibits Smo. In addition, the effect of cyclopamine on their survival was line-specific, in contrast to immediate gli inactivation by GANT61. Thus, our data indicate that the total activity of the gli proteins can be maintained both through Smo and independently of it, for example, due to deficient activity of protein kinase A [16]. Therefore, these results explain the contradiction in previous data on the sensibility of glioma cells to the regulation through the Shh signaling pathway [46, 56].

The ratio between full-length and truncated forms of gli reflects the regulation of gli activity by Shh signaling pathway through suppression of a partial cleavage of gli2 and gli3, and their accumulation at the tip of primary cilia [12, 13]. We found that some lines exhibited both the expression of gli target genes, including *GLI1*, and a high level of the gli3 truncated form, Gli3R, which is a transcriptional repressor of the gli target genes. In addition, the ratio of the Gli3R and Gli3FL levels in these lines was the similar or even higher than that in the normal adult brain tissue, where expression of the gli target genes was not detected. Since gli3 is intensely subjected to partial cleavage, its accumulation in the primary cilium, where this protein becomes a transcriptional activator, should be significantly reduced. However, *GLI3* knockdown showed that gli3 acted in glioma cells more as an activator, than a repressor of transcription, despite the high level of Gli3R. Based on these data, we assume that Gli3R only attenuates transcription of gli target genes, but does not prevent it, probably contributing to glioma cell heterogeneity.

## Conclusions

Thus, the gli transcription factors show abnormal activity in high-grade glioma cells, maintaining the expression of genes, determined the stem cell state, and contributing to glioma cell survival. At the same time, our data suggest that the total activity of gli can be maintained not only through Smo, but also independently of it, and gli can initiate the transcription of their target genes even despite a high level of the transcriptional repressor Gli3R.
